# Magnetic Resonance Imaging in Studying Schizophrenia, Negative Symptoms, and the Glutamate System

**DOI:** 10.3389/fpsyt.2014.00032

**Published:** 2014-04-03

**Authors:** Oliver Gruber, Antonella Chadha Santuccione, Helmut Aach

**Affiliations:** ^1^Center for Translational Research in Systems Neuroscience and Psychiatry, Clinic for Psychiatry and Psychotherapy, University Medical Center Göttingen, Göttingen, Germany; ^2^Medical Affairs – Psychiatry, Roche Pharma AG, Grenzach-Wyhlen, Germany

**Keywords:** schizophrenia, glutamate, negative symptoms, cognitive deficits, neuroimaging biomarkers, stratified therapy

## Abstract

Schizophrenia is characterized by positive, negative, and cognitive symptoms. While positive symptoms occur periodically during psychotic exacerbations, negative and cognitive symptoms often emerge before the first psychotic episode and persist with low functional outcome and poor prognosis. This review article outlines the importance of modern functional magnetic resonance imaging techniques for developing a stratified therapy of schizophrenic disorders. Functional neuroimaging evidence on the neural correlates of positive and particularly negative symptoms and cognitive deficits in schizophrenic disorders is briefly reviewed. Acute dysregulation of dopaminergic neurotransmission is crucially involved in the occurrence of psychotic symptoms. However, increasing evidence also implicates glutamatergic pathomechanisms, in particular *N*-methyl-d-aspartate (NMDA) receptor dysfunction in the pathogenesis of schizophrenia and in the appearance of negative symptoms and cognitive dysfunctions. In line with this notion, several gene variants affecting the NMDA receptor’s pathway have been reported to increase susceptibility for schizophrenia, and have been investigated using the imaging genetics approach. In recent years, several attempts have been made to develop medications modulating the glutamatergic pathway with modest evidences for efficacy. The most successful approaches were those that aimed at influencing this pathway using compounds that enhance NMDA receptor function. More recently, the selective glycine reuptake inhibitor bitopertin has been shown to improve NMDA receptor hypofunction by increasing glycine concentrations in the synaptic cleft. Further research is required to test whether pharmacological agents with effects on the glutamatergic system can help to improve the treatment of negative symptoms in schizophrenic disorders.

Neuroimaging techniques have been developed as important tools to investigate brain dysfunctions that underlie psychiatric disorders. In particular, modern functional magnetic resonance imaging (fMRI) holds the promise to provide neurofunctional biomarkers for improved diagnosis, prognosis, and optimized treatment of mental disorders [e.g., Ref. (1–4)]. In this brief, not exhaustive review, we will exemplify this translational neuroimaging research by focusing on schizophrenia and current challenges to advancing therapeutic approaches for this heterogeneous diagnostic category. First, the importance of negative symptoms and cognitive deficits for successful treatment of schizophrenic disorders will be highlighted, and an overview will be given on the neural correlates of these symptom domains as revealed by fMRI studies. Second, neuroimaging studies of glutamate levels and genetic risk factors pointing to an important role of glutamate–dopamine interactions in the pathophysiology of schizophrenic disorders will be briefly reviewed and will be related to recent findings of pharmacological and animal studies. In the final part of this review, we will present current approaches to develop therapeutic strategies that target the glutamatergic pathway in schizophrenic disorders.

## “Psychopathophysiology” of Schizophrenic Disorders and the Importance of Negative Symptoms and Cognitive Deficits

In traditional psychiatry, mental disorders are diagnosed and classified on the basis of more or less specific psychopathological symptoms in the course of the disorder. Numerous recent findings from modern systems neuroscience and molecular neuroscience strongly suggest that diagnoses made on the basis of psychopathological criteria do not represent “natural disease entities” in the sense of diseases with uniform pathogenesis and pathology [see Ref. (1, 3–8)]. Therefore, schizophrenia represents a group of mental disorders that share more or less characteristic psychopathological symptoms, such as verbal hallucinations, delusions, and formal thought disorders.

During the last two decades, modern brain imaging techniques have allowed for the first time scientific investigations into the neural correlates of different symptom dimensions that characterize schizophrenic disorders. These studies of pathophysiological processes that underlie different psychopathological symptoms and syndromes (an approach that may be termed “psychopathophysiology”) are briefly reviewed in the following.

Positive symptoms of schizophrenic disorders such as verbal hallucinations (“hearing voices”) and delusional symptoms are certainly the most impressive characteristics of schizophrenic disorders. Nevertheless, much evidence has been provided that negative symptoms and cognitive disturbances are more strongly associated with the long-term functional outcome of patients suffering from schizophrenia than positive symptoms (9–14). Cognitive deficits are even present at first episode and remain relatively constant over the course of illness (15–17). In contrast to the dopamine model of schizophrenia, glutamatergic theories of schizophrenia account for negative symptoms and cognitive dysfunction as well, and may therefore lead to new treatment approaches specifically targeting the unmet medical need to improve negative symptomatology and cognitive deficits (18).

### Neural correlates of positive symptoms in schizophrenic disorders

The positive symptoms of schizophrenic disorders particularly include auditory–verbal hallucinations (“hearing voices”) and delusional symptoms like paranoia. As regards auditory–verbal hallucinations, a number of functional brain imaging studies have been performed in the past. Overall, findings of these studies appear quite heterogeneous. However, a finding that has been replicated several times is overactivation of the superior temporal gyrus during experimental phases in which the patients exhibited the symptom of hearing voices [e.g., Ref. (19–21)]. In most of these studies, this activation of auditory association cortices was associated with additional activations in other brain regions. For instance, some of these studies also reported an increased brain activity in Broca’s area, the anterior cingulate cortex, the hippocampus, and the amygdala [e.g., Ref. (20)]. Some of these findings have also been confirmed on the meta-analytical level. Jardri et al. (22) confirmed that phases of “hearing voices” are associated with increased activity in Broca’s area, anterior insula, precentral gyrus, frontal opercular cortex, middle and superior temporal gyrus, inferior parietal lobule as well as in hippocampus and parahippocampal gyrus. It has to be noted, however, that patients suffering from this kind of intermittently occurring auditory–verbal hallucinations are probably not representative for most types of schizophrenic disorders in which the hallucinations persist for longer time periods. A second meta-analysis by Kühn and Gallinat (23) came to the conclusion that a current psychopathological state of experiencing auditory–verbal hallucinations may be associated with abnormal activation of brain regions that are also involved in speech production (e.g., Broca’s area), whereas a subgroup of schizophrenic patients that exhibits the symptom of auditory–verbal hallucinations (in comparison to a subgroup without “life-time diagnosis” of auditory–verbal hallucinations) may be particularly characterized by abnormal brain activation in areas involved in speech processing and, more generally, the processing of auditory stimuli. In another, qualitative and quantitative review, Goghari et al. (24) showed an association between positive symptoms, in particular ideas of persecution, with the activity in medial prefrontal cortex, amygdala, hippocampus, and parahippocampal gyrus.

### Neural correlates of negative symptoms in schizophrenic disorders

Negative symptoms of schizophrenic disorders are usually defined as symptoms representing a qualitative and/or quantitative reduction of mental capacities or qualities of experience. This class of symptoms is relatively heterogeneous and traditionally includes the five “A”s, which are affective flattening, apathy (reduced drive), anhedonia, asociality (social withdrawal), and alogia (impoverishment of thought) [e.g., Ref. (25)]. Early studies on the structural correlates of negative symptoms in schizophrenic disorders have shown gray matter reduction in temporal, medial frontal, insular, and hippocampal regions [e.g., Ref. (26)]. On the other hand, evidence on brain structural correlates of negative symptoms is very heterogeneous as there are also several studies that failed to find any correlation of brain volumes to negative symptoms in schizophrenia [e.g., Ref. (27–30)].

The development of functional neuroimaging techniques like PET and fMRI led to an increasing number of studies on the neurofunctional correlates of negative symptoms. Several studies have shown a reduced activation of the prefrontal cortex in schizophrenic patients with negative symptoms (25, 31–33). This principal finding of “hypofrontality” associated with negative symptoms has been confirmed by later studies though for different subregions of the prefrontal cortex. For example, in a study using memory retrieval tasks, Heckers et al. (34) found a significantly reduced recruitment of left frontal cortex (Brodmann area 44/9) in schizophrenic patients with deficit syndrome (i.e., patients with negative symptoms as primary and enduring features) as compared to both schizophrenic patients without deficit syndrome and healthy controls. Using auditory working memory tasks (*n*-back tasks), Menon et al. (35) found an inverse correlation of negative symptoms with activation in the frontal opercular cortex and in the right DLPFC. In contrast to that, another experiment using the *n*-back task (36) reported a correlation of activation deficits in the DLPFC with disorganization symptoms rather than with negative symptoms.

A number of functional neuroimaging studies have also reported associations of negative symptoms with activation in other brain regions including temporal cortices and the ventral striatum. For instance, Tamminga et al. (37) found both limbic system abnormalities and neocortical alterations associated with the deficit syndrome. A later series of studies have demonstrated a significant correlation of activation in temporal cortices with negative symptoms (38–40). Using the monetary incentive delay task, which activates the reward system, Juckel et al. (41) found a correlation of diminished ventral striatal activation with negative symptoms in schizophrenic patients. In another study by Simon et al. (42), a ventral striatal activation during reward anticipation was negatively correlated with symptoms of apathy, while activation during receipt of reward was negatively correlated with severity of depressive symptoms. Finally, in a recent systematic review and meta-analysis of 25 fMRI studies on schizophrenic symptomatology, Goghari and colleagues (24) have confirmed a relationship of negative symptoms with the functioning of the ventrolateral prefrontal cortex and the ventral striatum.

### Neural correlates of cognitive dysfunctions in schizophrenic disorders

Traditionally, psychiatric diagnosis of schizophrenic disorders is based on psychopathological, particularly positive and negative symptoms. Over the last few decades, the advent of modern experimental neuropsychological and functional neuroimaging techniques has switched the focus of interest toward brain dysfunctions in the domains of cognitive, emotional, and motivational processes that are highly prevalent in schizophrenic disorders. Especially cognitive deficits are of major interest as they have been proposed to represent core deficits of schizophrenic disorders [e.g., Ref. (43, 44)]. These core cognitive deficits of schizophrenic disorders include deficits in working memory, executive functions, episodic memory, and social cognitions.

Deficits in working memory in schizophrenic disorders have been found to be associated with dysfunctions of prefrontal cortices, especially of the dorsolateral prefrontal cortex, of the deep fronto-opercular cortex, and of the anterior cingulate cortex [e.g., Ref. (45–50)]. In the last few years, there have also been several reports of a disturbed connectivity between these prefrontal areas and the medial temporal lobe, particularly the hippocampus [e.g., Ref. (51, 52)].

Executive function is a construct that encompasses a variety of sub-functions [see for example Ref. (53, 54)], among them selective attention, background monitoring of the environment for potentially significant sensory events [e.g., Ref. (55)], and the adaptation of behavior to changing environmental conditions [e.g., Ref. (56–60)]. Patients with schizophrenic disorders exhibit multiple dysfunctions in these areas of executive control mechanisms that are associated with reduced activity in the posterior frontal medial cortex and the inferior frontal junction area (IFJA; a cortical subregion at the intersection of the precentral sulcus and the inferior frontal sulcus) (61, 62) as well as with abnormally increased activations in brain stem nuclei and the ventral striatum (63).

Episodic memory deficits in schizophrenic disorders have been found to be associated firstly with dysfunction of the extended hippocampal formation (including the hippocampus proper and the surrounding medial temporal structures) [e.g., Ref. (64)], and secondly with dysfunctions of prefrontal cognitive control mechanisms that are involved in encoding and retrieval processes (65).

Disturbed social cognitions in schizophrenic disorders include particularly impaired recognition of facial emotional expressions and reduced theory-of-mind capacities. Neuroimaging studies have revealed that deficits in these domains of social cognitions are associated, firstly, with reduced activation in the amygdala and the fusiform gyrus (66) and, secondly, with reduced activity in the fronto-median cortex, in the temporo-parietal junction cortex, and the amygdala–hippocampus complex [e.g., Ref. (67)]. Disturbed functional coupling between prefrontal areas and the amygdala has also been reported as a correlate of impaired emotional regulation mechanisms (68).

Taken together, neuroimaging studies on the neural correlates of different (positive, negative, cognitive) symptom domains in schizophrenic disorders suggest the involvement of different lateral and medial prefrontal, temporal (particularly including hippocampus and amygdala), and subcortical (in particular ventral striatum as part of the dopaminergic reward system) brain regions in the occurrence of these symptoms.

## Pathogenesis, Pathophysiology, and Treatment of Schizophrenic Disorders: The Role of Glutamate–Dopamine Interactions

**Figure 1:**
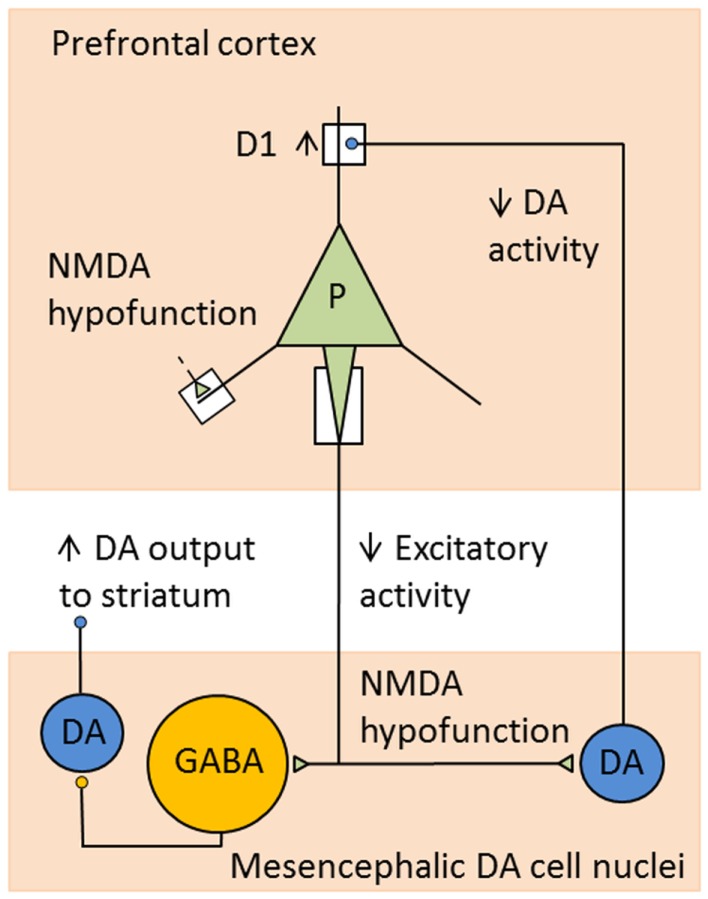
**Example for consequences of NMDA receptor hypofunction in glutamatergic–dopaminergic circuits**. Hypofunction of NMDA receptors mediating excitatory inputs to prefrontal pyramidal cells in schizophrenia leads to decreased activity in cortical excitatory projections to mesencephalic DA cell nuclei. This results in decreased activity of DA neurons projecting to the DLPFC and increased activity of DA cells projecting to the striatum, as a consequence of decreased stimulation of GABA interneurons. Reduced DA levels in DLPFC lead to compensatory, but functionally insufficient, upregulation of D1 receptors [adapted from Lewis and Gonzalez-Burgos (72)].

It is well-established that acute dysregulation of dopaminergic (and glutamatergic) neurotransmission is crucially involved in the occurrence of psychotic symptoms, whereas more chronic cellular neuropathology may be responsible for the development of cognitive deficits and negative symptoms (69). In more recent years, classical elements of the dopamine hypothesis such as the overactive mesolimbic dopamine system and a reduced mesocortical dopamine turnover are considered rather as a “final common pathway” (70), i.e., as the expression of upstream pathophysiological changes. In particular, it is postulated that *N*-methyl-d-aspartate (NMDA) receptor dysfunction may lead to dopaminergic dysregulation in schizophrenic disorders through a complex interaction between glutamatergic and dopaminergic, but also GABA-ergic mechanisms. Schwartz and co-workers (71), for example, explain both positive and negative symptoms of schizophrenia with dysfunctions of NMDA-glutamatergic synapses. Via effects on various complex circuits including GABA-ergic interneurons, these dysfunctions ultimately result both in hyperfunction of the mesolimbic dopamine system leading to positive symptoms, and also in hypofunction of the mesocortical dopamine system associated with negative symptoms and cognitive dysfunctions (Figure 1). Such pathomechanisms in functional interactions between prefrontal cortices and brain stem nuclei, in particular the ventral tegmental area (VTA), the striatum, and the thalamus, may be influenced by predisposing and/or protective genetic variants that exert effects on the glutamatergic synapse. Thus, the glutamatergic hypothesis is also compatible with our knowledge about the effects of susceptibility genes of schizophrenia (see below).

Modern pharmacological and animal research approaches are increasingly focusing on the interactions between the dopaminergic and glutamatergic system, especially within fronto-striato-thalamo-frontal loops and in the interactions between frontal cortex, hippocampus, nucleus accumbens, and VTA. Within and between these brain regions, the dopamine and the glutamate system interact in a very complex way, and dysfunctions in these interactions are a central pathomechanistic explanation for the development of schizophrenic disorders [e.g., Ref. (73, 74)]. Particularly, the hippocampus plays a major role in regulating the dopaminergic reward system. Animal studies have shown that increased activity of the ventral hippocampus (subiculum) leads to increased dopamine turnover in the ventral striatum (nucleus accumbens) (75, 76). Other recent findings suggest that this glutamatergically mediated effect of the subiculum on the nucleus accumbens further leads to increased GABA-ergic projection onto the ventral pallidum with reduced tonic activity of the pallidum and consecutive disinhibition of dopamine neurons in the VTA (77). In this way, hyperactivity of the ventral hippocampus observed in schizophrenic disorders could lead to overstimulation and hyperactivity of dopaminergic VTA neurons which may account for various symptoms, in particular for delusional phenomena. Further support for this theory is provided by studies using the MAM animal model of schizophrenia which have shown that the relevant pathophysiological changes such as VTA hyperactivity and increased response to amphetamines are no longer present after inactivation of the subiculum. This indicates that the subiculum is necessary to induce hyperdopaminergic states in this animal model (78).

The pathophysiological role of glutamatergic disbalances has also been investigated *in vivo* in patients with schizophrenia, for example using magnetic resonance spectroscopy (MRS). In very recent publications (79, 80), findings of such glutamatergic proton magnetic resonance spectroscopic imaging studies have been nicely reviewed, particularly with respect to their implications for drug discovery. Overall, neuroimaging studies support the current glutamate model of schizophrenia by suggesting a hypofunction of the NMDA receptor. In particular, proton magnetic resonance spectroscopic (1H-MRS) studies have provided evidence for altered levels of glutamate and glutamine in the medial prefrontal cortex and in the basal ganglia in early-stage, drug-naïve, or drug-free schizophrenia patients.

Some studies with unmedicated patients with schizophrenia have reported elevated glutamatergic levels in the medial prefrontal cortex as compared to healthy controls (81–84). More precisely, a recent meta-analysis by Marsman and colleagues (79) indicated that it is glutamine which is increased in the frontal cortex in schizophrenic patients, whereas glutamate is reduced. Such an elevated glutamine/glutamate ration may result from either a deficiency in glutaminase, which converts glutamine into glutamate, or from NMDA receptor hypofunction which has also been shown to increase glutamine levels and decrease glutamate levels (79). Further, glutamate levels in the medial prefrontal cortex have been found to be associated with negative symptoms and worse global functioning and to be decreased in remitted patients as compared to non-remitted patients (85). Consistent with that, most studies comparing medicated patients with healthy control subjects reported unchanged glutamate levels in the medial prefrontal cortex (81, 86–92). The meta-analysis by Marsman and colleagues (79) provided additional support for a progressive decrease of frontal glutamate and glutamine in patients with schizophrenia possibly indicating a progressive loss of synaptic activity. Finally, particularly in first episodes schizophrenic patients, increased glutamatergic levels have also been reported in the basal ganglia (93–95), and they appear to decrease to normal levels during antipsychotic treatment with risperidone (94).

Over the last 10–15 years, numerous potential susceptibility genes of schizophrenia have been identified, among them COMT, dysbindin-1, neuregulin-1, RGS4, GRM3, and DISC1. Many of these candidate genes have been shown to influence dopaminergic and/or glutamatergic neurotransmission, and effects on neuroplastic processes and particularly on synaptogenesis have also been reported. Imaging genetics is still a relatively novel approach that, however, has already made substantial contributions to our knowledge about genetic effects on brain structure and function. Early studies, for example, demonstrated the influence of variants in the COMT gene on working memory-related prefrontal activation (96) and on the functional interplay between dopamine synthesis in the midbrain and prefrontal function (97). Although the evidence for an association between the COMT gene and schizophrenia is not unequivocal, these findings nevertheless have high biological plausibility insofar as the influence of the COMT gene on the dopaminergic tone in the prefrontal cortex has been convincingly demonstrated (98). Further studies on the COMT genotype have shown more complex haplotype effects on prefrontal cerebral activations (99) and on gene–gene interactions between COMT and other genes such as RGS4, G72, DISC1, and GRM3 (49, 100, 101). Especially the latter finding is consistent with a role of glutamate–dopamine interactions in the pathophysiology and pathogenesis of schizophrenic disorders.

The number of genome-wide association studies between gene variants and diseases has markedly increased over the last few years due to the availability of modern chips. This has also inspired imaging genetics studies as genome-wide confirmed risk variants have also been investigated for their effects on brain structure and function. Two examples of this are the zinc finger protein 804A (ZNF804A), the function of which has not yet been more closely characterized, but which showed a genome-wide significant association with schizophrenia and also with bipolar disorder (102), as well as the CACNA1C gene, which was first discovered as a risk gene for bipolar disorder, but later also for schizophrenia (103). Studies on the ZNF804A polymorphism have shown an effect on the connectivity between the prefrontal cortex and the hippocampus (104–106). Effects of the CACNA1C gene have been reported with regard to activation of the hippocampus and the subgenual ACC (107) as well as activation of the amygdala during reward and fear recognition paradigms (108, 109).

Taken together, the studies summarized here support the important pathophysiological role of glutamate in schizophrenia and encourage further development of therapeutic strategies that target the glutamatergic pathway in schizophrenia.

## Therapeutic Strategies Targeting the Glutamatergic Pathway in Schizophrenia

A valid treatment for positive symptoms, based on the use of antipsychotic agents and their main capacity to modulate the dopaminergic system, is currently available for schizophrenia. However, antipsychotics are less effective in reducing negative symptoms or in ameliorating cognitive dysfunctions (110–112). Based on the novel findings that the glutamatergic system plays an important role in the pathogenesis of schizophrenia, several attempts have been made to identify drugs which, by modulating this system, could improve negative symptoms and cognitive dysfunction (113). Pharmacological targets are different types of glutamate receptors, which interact in a complex not yet fully understood way within glutamatergic synapses. These receptors include both ionotropic receptors [NMDA, α-amino-3-hydroxy-5-methyl-4-isoxazolepropionic acid (AMPA), and kainate receptors] and metabotropic glutamate receptors (mGluRs) [reviewed in Ref. (114)]. AMPA and kainate receptors are fast gating by glutamate binding and permeable for Na^+^ and K^+^ ions, whereas NMDA receptors exhibit a slow gating kinetic and need a predepolarization of the neuronal membrane for activation by glutamate binding. This predepolarization occurs when vicinal AMPA receptors are frequently activated and leads to the release of a blocking Mg^2+^ ion from the NMDA receptor. Activated NMDA receptor channels are not ion specific and pass Ca^2+^, Na^+^, and K^+^ ions, which leads not only to further depolarization but also intracellular processes making the synapse more sensible for signals from upstream and sending stronger signals downstream. This so-called long-term potentiation (LTP) provides the basis for synaptic and dendritic proliferation or pruning, learning, and memory (115).

Metabotropic glutamate receptors are G protein-coupled receptors influencing intracellular metabolic processes and are present on presynaptic and postsynaptic neurons as well as on glial cells near glutamatergic synapses. Currently, eight mGlu receptors are known of which the mGlu 2/3 receptors are investigated as targets for schizophrenia therapy, because they regulate presynaptic glutamate secretion.

After successful completion of preclinical trial programs of pharmacodynamic activity and safety, potential compounds are investigated in escalating clinical study programs. Phase 1 studies are open label, single to multiple dose trials with healthy volunteers, exploring pharmacokinetic and first safety characteristics. Phase 2 studies are explorative or proof-of-concept studies that are usually controlled and blinded with small to medium numbers of patients, and designed for dosis finding and first in patient proof of safety and efficacy. Phase 3 studies are large scale, double blinded controlled studies to confirm safety and efficacy. Some of the following described trial results are not yet published in peer-reviewed journals, and we had to quote congress presentations or company statements.

### Compounds enhancing NMDA receptor function

Since the activation of the NMDA receptor also requires glycine as a co-agonist, the glycine binding site at the NMDA receptor is regarded as a promising pharmacological target to enhance its activity, thereby minimizing the risk of excitotoxicity that is associated with direct overactivation of the glutamate binding site (116). A first review of clinical trials published until 2003 with agonists of the glycine binding site of the NMDA receptor including glycine, d-cycloserine, and d-serine reported moderate effect sizes for glycine and d-serine on negative symptoms, but no effect of d-cycloserine (117). It included 358 small randomized trials with 6–51 participants and a maximum duration of 12 weeks. Up to now, the amount of data has grown confirming these early results. In almost all trials, the compounds were used as adjunctive treatment to regular antipsychotic therapy, and generally no effect was reported in patients taking clozapine.

Several clinical trials have been conducted using glycine, a substance that is endogenously produced and that can act as a co-agonist of NMDA receptors binding at the glycine modulatory site. The results of these studies suggest that glycine can significantly improve symptoms of schizophrenia (118), including negative symptoms, although there are also negative and equivocal studies (119).

d-Cycloserine is a selective co-agonist of NMDA receptors containing the NR2C subunit, and these receptors are involved in fear conditioning and memory consolidation (120). When used as a single dosis in rodents, d-cycloserine led to enhanced memory consolidation of novel information (120). In an exploratory clinical trial, once weekly dosing of d-cycloserine augmentation over 8 weeks did not improve cognitive symptoms but reduced negative symptoms and delusion severity in stable schizophrenic patients medicated with a range of different antipsychotics excluding clozapine (121).

d-Serine, another agonist of the glycine modulatory site within the NMDA receptor, has been shown in clinical trials to be capable of ameliorating several symptom domains in schizophrenia (122–124).

### Glycine transporter-1 inhibitors

Sarcosine is a potent natural glycine transporter-1 inhibitor (GlyT-1) (125). The inhibition of this transporter leads to increased levels of glycin in the synapsis and consequently enhanced NMDA receptor activation, which may represent a possible treatment mechanism for schizophrenic disorders in which a hypofunction of NMDA receptors is present (126). As recently shown, sarcosine may reduce negative symptoms in acutely ill schizophrenia patients receiving atypical antipsychotics, being more effective than the NMDA/glycine site agonist d-serine (125).

**Figure 2:**
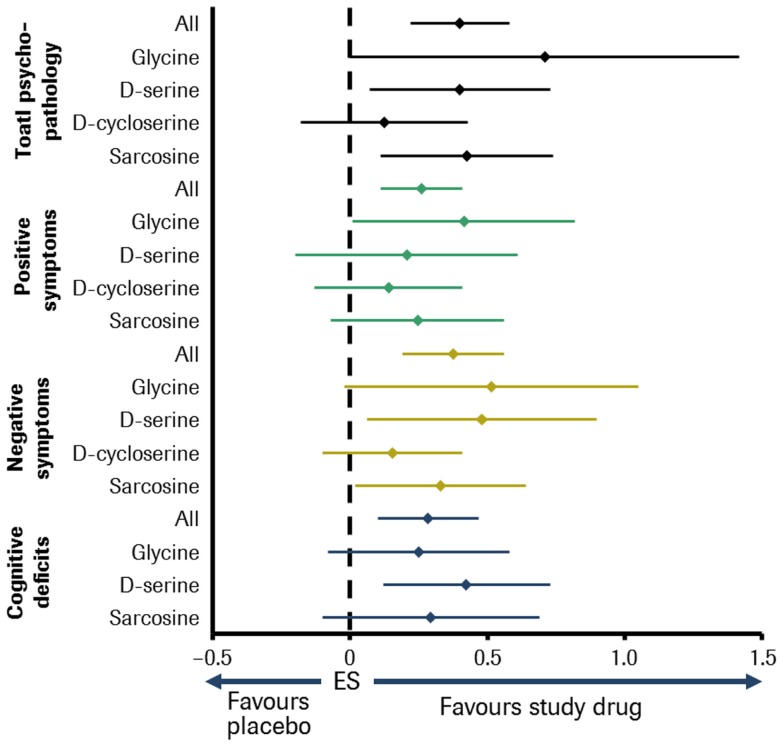
**Meta-analysis of double-blind, placebo-controlled studies of small-molecule NMDA receptor enhancers in patients with schizophrenia**. Sample size: 26 studies comprising about 800 patients. Effect size (ES) for glycine, d-serine, d-cycloserine, sarcosine, and all in different symptom domains of schizophrenia [adapted from Tsai and Lin (118)].

A meta-analysis including all abovementioned molecules found that, overall, the NMDA-enhancers were effective against most symptom domains of schizophrenia. Glycine, d-serine, and sarcosine significantly improved multiple symptom domains, whereas no symptom domain was improved by d-cycloserine. Furthermore, glycine, d-serine, and sarcosine were found to be superior to d-cycloserine in improving overall psychopathology [Ref. (118); see Figure 2]. However, these compounds have individual disadvantages to be developed to drugs licensed for long-term use. Glycine is fast metabolized and passes the blood–brain barrier poorly. So it has to be applied with daily doses up to 60 g. Although d-serine is substantially metabolized, daily doses of 30–120 mg/kg were effective. But there is concern about nephrotoxicity at higher doses, although no significant adverse events have yet been observed at doses of ≤4 g/day (127). According to Sreekumar et al. (128), sarcosine might play a role in aggravating prostate cancer progression.

Bitopertin is a GlyT-1 inhibitor that increases levels of the glycine neurotransmitter by inhibiting its reuptake from the synaptic cleft. Preclinical evidence showed that this molecule is capable of ameliorating the symptoms of schizophrenia in animal models (129, 130). These preclinical findings encouraged a double blinded placebo-controlled phase IIb clinical trial which showed that adjunctive treatment with bitopertin in stable patients with predominant negative symptoms was capable of ameliorating negative symptoms and improving general clinical status (131). Currently, several phase III studies are underway with the hope that bitopertin may help in the treatment of currently unsatisfyingly responding stages of schizophrenia.

A major goal for future research combining psychopharmacology and modern functional neuroimaging techniques would be to understand how these molecules modulate the activity of pathophysiologically relevant neural structures as outlined above. Such studies could, for example, provide important information on whether these pharmacological agents can be successfully used to treat patient subgroups that are characterized by specific symptoms of schizophrenia.

### AMPA receptor modulators

As described above, the fast trafficking of AMPA receptors in the synaptic cleft has an impact on NMDA receptor-mediated LTP and depression. These intracellular mechanisms influence synaptic strength and therefore constitute the basis of learning and memory (132). Therefore, modulation of AMPA activity could lead to amelioration of cognitive dysfunction in schizophrenia. To this aim and based on preclinical evidences, two molecules have been used in schizophrenia clinical trials: piracetam (133) and CX516 (121, 134).

Piracetam augmentation of haloperidol was capable of improving psychotic symptoms in schizophrenia, but had no effect on PANSS (133). Because only 30 patients (all receiving haloperidol) completed the placebo-controlled trial, more scientific evidence is needed to support such an effect. Trials with CX156 led to controversial results. In a small study, CX156 improved cognitive functions and negative symptoms in schizophrenic patients when compared to patients treated with clozapine (134). However, a larger study was unable to show any effect of CX516 on cognition or negative symptoms when compared to controls (135). Taken together, there is only little evidence about these molecules and their therapeutic effect.

### mGlu receptor modulators

In contrast to the concept of improving symptoms of schizophrenia with ampakines, a line of evidence points to an overactivation of AMPA synapses in the prefrontal cortex downstream of NMDA receptor hypofunction (136). NMDA receptor blockade on GABA-ergic interneurons reduces inhibition of pyramidal cells and leads to excessive glutamate release in AMPA receptor synapses in the prefrontal cortex (137). Metabolic glutamate receptors 2 and 3 (mGlu 2/3) facilitate a feedback regulation of synaptic glutamate release (138). Consequently, mGlu 2/3 enhancing agents were developed to delimit pathologically enhanced glutamate release. In schizophrenia, clinical trials were conducted with the mGluR2/3 agonist, pomaglumetad methionil (LY2140023) and the mGlu2 positive allosteric modulator, ADX71149.

Pomaglumetad methionil was investigated as monotherapy in three clinical trials. At first, a phase 2 proof-of-concept trial showed significant improvement of positive and negative symptoms versus placebo (139). In a following phase 2 dose ranging trial, all of the four investigated dosing groups did not differ from placebo (140), which was also the case for a phase 2 trial comparing pomaglumetad methionil to olanzapine and a placebo group. In this trial, both active treatment groups did not separate regarding efficacy and safety parameters from the placebo group (141).

After demonstrating an augmentation of the efficacy of atypical antipsychotics in preclinical trials, a phase 1 study was conducted to prove the safety of the combination of pomaglumetad methionil with four different second generation antipsychotics in healthy subjects (142). A following placebo-controlled phase 2 study tested the substance as adjunctive to standard of care in patients with prominent negative symptoms of schizophrenia. This trial did not indicate a significantly greater reduction of negative symptoms or an improvement of secondary efficacy endpoints over placebo (143). Based on these results, a phase 3 study started in the meantime was stopped.

The mGluR2 selective positive allosteric modulator ADX71149 is co-developed by Addex Therapeutics (144) and Johnson & Johnson, who code it JNJ40411813. Results of a randomized placebo-controlled phase 2 study evaluating the safety, tolerability, and exploratory efficacy of the compound given in two different doses as adjunctive to an ongoing antipsychotic medication were reported at the 2013 annual meeting of the American Psychiatric Society. The study population comprised three groups: patients with residual negative symptoms, patients with residual positive symptoms, and patients with insufficient response to clozapine treatment. Tolerability results suggest that dose titration may be beneficial. An efficacy signal seen in the negative symptoms subgroup treated with the lower dose suggests this population warrants further evaluation in a formal proof-of-concept study (144, 145).

## Conclusion and Future Perspectives

In this brief review article, we have summarized recent findings from genetic, animal, and functional neuroimaging studies that together point to an important role of glutamate–dopamine interactions within cortico-striato-thalamo-cortical (CSTC) loops, which are modulated by hippocampal and amygdala inputs, in the pathophysiology of schizophrenic disorders. These findings provide the empirical basis for the development of novel treatment approaches for schizophrenic disorders that target glutamatergic mechanisms. The new findings from animal studies may also inspire analogous clinical neuroimaging investigations of neurofunctional interactions within CSTC loops as well as of the dynamic effects of mediotemporal structures such as hippocampus and amygdala on these CSTC loops in the human brain. Here, the development of valid animal model-supported experimental paradigms is of major importance as it may allow for the targeted *in vivo* investigation of these pathomechanisms involved in the pathophysiology of schizophrenic disorders [e.g., Ref. (4)]. Such targeted investigations may enable a future stratification of the heterogeneous group of schizophrenic disorders into pathophysiologically more homogenous “natural disease entities” (146). Moreover, the development of neurofunctional MRI biomarkers for sub-classification of patient groups and prediction of individual treatment responses may generally play an important role in a future individualized medicine in psychiatry [e.g., Ref. (4)].

## Conflict of Interest Statement

Oliver Gruber was honorary speaker for the following companies: Astra Zeneca, Bristol Myers Squibb, Janssen Cilag, Lilly, Lundbeck, Otsuka, Servier. Oliver Gruber has been invited to scientific congresses by Astra Zeneca, Janssen Cilag, Pfizer, Servier. Oliver Gruber has received a research grant from Servier and a publication grant from Roche. Antonella Chadha Santuccione and Helmut Aach are full employes of Roche Pharma AG, Grenzach, Germany.
